# The controversy of patellar resurfacing in total knee arthroplasty: Ibisne in medio tutissimus?

**DOI:** 10.1007/s00167-012-1985-7

**Published:** 2012-04-08

**Authors:** Oliver S. Schindler

**Affiliations:** Bristol Arthritis & Sports Injury Clinic, St Mary’s Hospital, Upper Byron Place, Clifton, Bristol BS8 1JU UK

**Keywords:** Patella, Patellar resurfacing, Total knee arthroplasty, Anterior knee pain, Femoral component design, National joint register

## Abstract

Early arthroplasty designs were associated with a high level of anterior knee pain as they failed to cater for the patello-femoral joint. Patellar resurfacing was heralded as the saviour safeguarding patient satisfaction and success but opinion on its necessity has since deeply divided the scientific community and has become synonymous to topics of religion or politics. Opponents of resurfacing contend that the native patella provides better patellar tracking, improved clinical function, and avoids implant-related complications, whilst proponents argue that patients have less pain, are overall more satisfied, and avert the need for secondary resurfacing. The question remains whether complications associated with patellar resurfacing including those arising from future component revision outweigh the somewhat increased incidence of anterior knee pain recorded in unresurfaced patients. The current scientific literature, which is often affected by methodological limitations and observer bias, remains confusing as it provides evidence in support of both sides of the argument, whilst blinded satisfaction studies comparing resurfaced and non-resurfaced knees generally reveal equivalent results. Even national arthroplasty register data show wide variations in the proportion of patellar resurfacing between countries that cannot be explained by cultural differences alone. Advocates who always resurface or never resurface indiscriminately expose the patella to a random choice. Selective resurfacing offers a compromise by providing a decision algorithm based on a propensity for improved clinical success, whilst avoiding potential complications associated with unnecessary resurfacing. Evidence regarding the validity of selection criteria, however, is missing, and the decision when to resurface is often based on intuitive reasoning. Our lack of understanding why, irrespective of pre-operative symptoms and patellar resurfacing, some patients may suffer pain following TKA and others may not have so far stifled our efforts to make the strategy of selective resurfacing succeed. We should hence devote our efforts in defining predictive criteria and indicators that will enable us to reliably identify those individuals who might benefit from a resurfacing procedure. *Level of evidence* V.

## Introduction

**Fig. 1:**
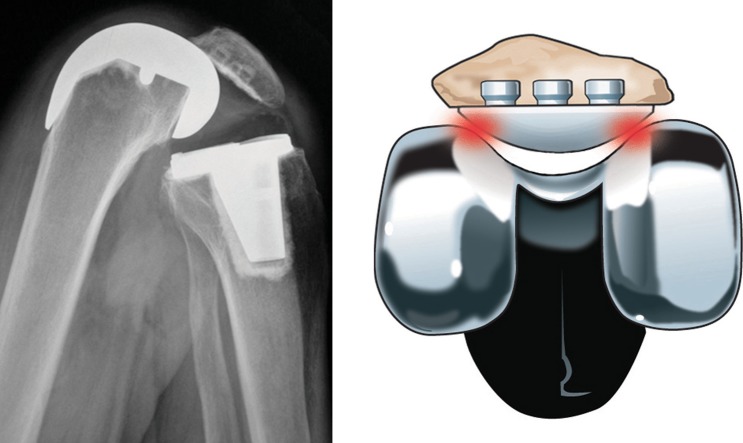
High patello-femoral reaction forces occur during knee flexion beyond 90°, when the patellar component leaves the trochlea groove, straddling the intercondylar notch, and contact areas decrease dramatically [124]

The patello-femoral articulation is exposed to the highest stresses within the locomotor system with recorded peak levels of up to 20 × body weight [123, 137, 155]. It is therefore not surprising that in 1977, Matthews et al. [86] expressed the view that ‘high patello-femoral load values, small patello-femoral contact areas, and resultant high stress magnitudes indicate the need for caution in the design and development of a patello-femoral component for total joint replacement prosthesis’. Their statement remains applicable even today, as retrieval analysis of patella components and the significant failure rate of metal-backed patella designs in the 1980s underscore the extreme mechanical environment in which these implants are expected to perform [8, 27, 59, 60, 116, 124, 144] (Fig. 1).

**Fig. 2:**
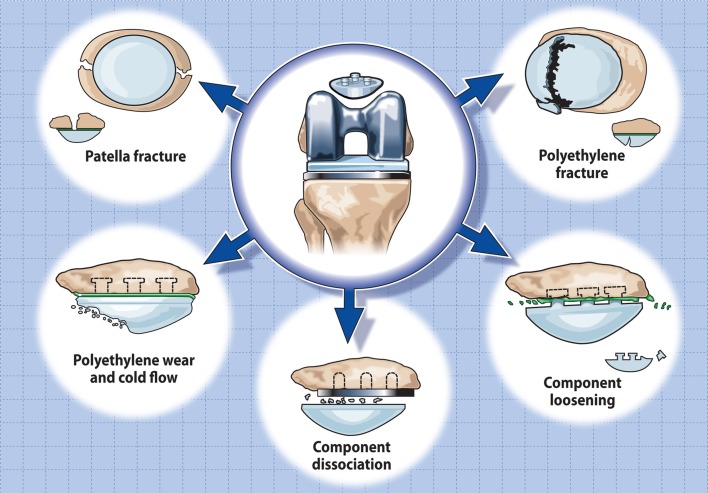
Common failure modes associated with patellar resurfacing

The earliest types of total knee arthroplasties were pure tibio-femoral replacements, primarily designed to treat severe axial deformities and intractable knee pain in patients affected by either tuberculosis or rheumatoid arthritis [47, 134, 150, 152]. They frankly ignored the patello-femoral joint, and associated patellar complications were often treated rather nonchalantly with patellectomy. Arthroplasty procedure at that time was seen as an alternative to arthrodesis and performed in patients of extremely low demand, where any improvement in pain relief or mobility level was considered a success [134, 152]. Increased patello-femoral complications and extensor mechanism failures raised awareness of the short comings of available knee implants failing to provide for normal patello-femoral function [63, 93, 131]. A case in point was the Duocondylar prosthesis which initially did not cater for the PFJ, providing disappointing results with a high level of patients suffering anterior knee discomfort [108]. Changes in femoral component design through the addition of a trochlear flange (Duopatellar design) improved clinical outcome dramatically by allowing the natural patella to articulate with the femoral component throughout the whole range of flexion [108, 109]. However, clinical results remained unpredictable and encouraged clinicians to experiment with replacement of the retro-patellar surface [2, 50, 51, 53, 54, 110]. In the 1980s, the patella was eventually removed from its Cinderella status and resurfacing was heralded as the saviour safeguarding patient satisfaction and success when replacing the knee. Amstutz even considered the term total knee arthroplasty a misnomer unless it incorporates the use of a patellar component [4]. Within a short period of time, patellar resurfacing was universally accepted as an integral part of total knee arthroplasty providing an improved level of patient satisfaction [65]. Over time, patellar resurfacing, however, became associated with complications specific to the patello-femoral joint which despite improvements in surgical technique and component design have not been eradicated (Fig. 2) [17, 68, 111, 113]. Omission of the patella on the other hand was seen to be responsible for an increase in the occurrence of anterior knee pain, which unfortunately failed in a large proportion of patients to respond to secondary resurfacing. The surgical community has hence become divided on the issue of how the patella is best served when performing total knee arthroplasty, and arguments for and against resurfacing have continued into the 21st century [1, 11, 18, 125, 128]. This article tries to address some of the questions surrounding the current controversy regarding patellar resurfacing and to balance the different points of view in an attempt to define what may be considered best medical practice.

## Pros and cons of patella resurfacing

In 1836, Malgaigne of Paris wrote ‘When one searches among the past or present authors for the origins of doctrines generally accepted today concerning dislocation of the patella, one is surprised to find among them such disagreement and such a dearth of facts with such an abundance of opinions’ [82]. Although focussing on a slightly different subject matter, Malgaigne’s view very much characterises the diversity of opinions expressed in the debate about the value of patella resurfacing in TKA, which according to Krackow has become analogous to topics of religion and politics [71]. For Robertsson, “the usefulness (or not) of the patellar button is mostly a matter of ‘belief’, and opinion builders (surgeons and representatives) have a good opportunity to influence this” [120].

Three basic treatment strategies pertaining to the use of patellar components have evolved so far: always to resurface, never to resurface, or to selectively resurface the patella. Clinicians who prefer patellar resurfacing claim reduced incidence of post-operative anterior knee pain (AKP), avoidance of secondary resurfacing, higher patient satisfaction, better overall function, and a low complication rate [14, 75, 110, 130, 151]. They also argue that the procedure is relatively inexpensive and not time-consuming when performed during standard TKA. The articulation between cartilage and metal is considered unphysiological, and prolonged exposure to high compressive forces is believed to cause cartilage erosion [42]. So far, however, no conclusive evidence exists that patellae affected by such changes become symptomatic [69, 75, 141]. The proportion of revisions attributable to the resurfaced patella has dropped over the past 25 years from almost 50 % in the 1980s to around 12 % today [17, 66, 132]. The prevalence of patello-femoral complications has also decreased significantly and currently remains at around 4–5 % [7, 13, 73, 91, 156].

Clinicians in support of non-resurfacing argue that clinical results between patients with and without resurfacing are broadly similar and that patellar resurfacing therefore represents an unnecessary step in performing a TKA. Other claims pertain to conservation of patellar bone, reduced likelihood of patellar osteonecrosis, more physiological patello-femoral kinematics, ability to withstand high patello-femoral forces especially in younger and more active patients without the concern of prosthetic wear or failure, and ease of resurfacing in case of recalcitrant AKP [1, 23, 37, 69]. Particular emphasis is generally placed on the avoidance of intra- and post-operative complications associated with patellar resurfacing which have been reported in 4–35 % of cases, even when using contemporary total knee designs, and which include patella mal-tracking and sub-luxation, component wear and loosening, patella fracture, extensor mechanism failure, and AKP [8, 31, 32, 68, 113].

The paradigm of selective resurfacing attempts to identify those individuals who are thought to have an improved clinical outcome with patellar resurfacing whilst avoiding potential complications associated with unnecessary resurfacing [1, 17, 55, 69, 70, 76, 107, 128, 135, 141]. Advocates of selective patellar resurfacing have based their decision on the presence of certain prerequisites pertaining mainly to patient-related and prostheses-related factors. A number of patient selection criteria which favour patella retention have been suggested and include patients below the age of 65, absence of AKP or crystalline disease, reasonably well-preserved retro-patellar cartilage (e.g. viable cartilage without evidence of eburnised bone or Outerbridge grade IV changes), anatomical normality (e.g. adequate patello-femoral congruence, normally shaped patella of adequate thickness), and normal patellar mechanics (e.g. central patellar tracking). Survival rates of up to 97.5 % at 10 years in non-resurfaced total knee arthroplasties have been reported when these selection criteria are applied [70].

Some argument exists about the indication of patellar resurfacing in patients affected by inflammatory arthropathies. Sledge and Ewald suggested that failure to resurface the patella in rheumatoid arthritis may allow continued release of sequestered antigen from the retained cartilage resulting in recurrent inflammation [136]. Concerns about an ongoing inflammatory process, however, have remained largely theoretical, and although various studies have recommended routine resurfacing on all patients with RA [7, 76, 107, 127], others have failed to notice any ill effects despite patellar retention [1, 13, 30, 36, 55, 97, 135].

**Fig. 3:**
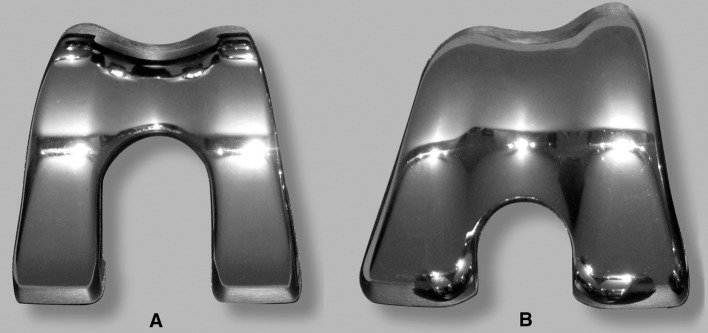
Two femoral components demonstrating design changes to improve patellar function. Unmodified Ortholoc^®^ femoral component with relatively patella unfriendly trochlea configuration (*right*) and modified Ortholoc^®^ femoral component (*left*) with asymmetrical, anatomic femoral groove, elevated lateral trochlea flange, and elongated trochlea groove (Arthroplasty components courtesy of Leo Whiteside and associates from the Missouri Bone and Joint Research Foundation, St Louis/MO, USA)

When resurfacing the patella, the surgeon is required to adhere to strict surgical principles in order to reproduce patellar thickness, preserve patellar blood supply, achieve appropriate positioning of all implant components, and balanced soft tissues to allow for central patellar tracking [71, 99, 122]. Prostheses-related factors are also critical to the success whether the patella remains resurfaced or not. The importance of femoral component design and its influence on patello-femoral performance has been highlighted by Theiss et al. [148] based on clinical results of two arthroplasty designs with distinct differences in trochlear geometry. A 14-fold decrease in patella-related complications was observed when using a patella-friendly design with an extended anterior flange, and a deeper and wider trochlea groove. The authors concluded that more proximal capture of the patella in a deeper groove with more gradual proximal-to-distal transition appeared advantageous in reducing patella morbidity. The group of Whiteside used an experimental model and was able to demonstrate that specific femoral design changes including deepening and distal extension of the trochlea groove improved patella tracking compared with an unmodified femoral component [158] (Fig. 3). The choice of prosthetic design with a patella-friendly femoral component has proven even more critical when the patella is left unresurfaced [12, 61, 81, 84, 85, 97, 147, 148]. Advocates of non-resurfacing hence favour femoral components of anatomically shaped trochlear configuration which attempt to provide a matching articulating surface to better accommodate the native patella.

## Complications associated with patellar resurfacing

**Fig. 4:**
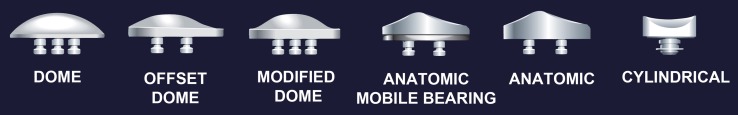
Commonly used types of patellar component design configurations [125]

The advent of patellar resurfacing inadvertently introduced a new and different set of complications to the clinician performing TKA (Fig. 2). Failures associated with the PFJ are multifactorial and may relate to patient selection (e.g. age, BMI), surgical technique or implant design (e.g. dome, anatomic, mobile bearing) (Fig. 4) [111, 112]. The most common reason for patellar complications and premature patellar failure, however, is surgical mismanagement or misjudgement and the consequences thereof. Patellar complications include post-operative patellar mal-tracking and instability, patellar fracture, polyethylene wear, component loosening and dissociation, soft tissue impingement, and extensor mechanism disruption. Component design, material choice and the manufacturing process also appear to have a significant effect on performance, longevity and potential complications. Cases in point are the high failure rate associated with metal backing of patellar components and the use of carbon fibre re-enforced ultra-high molecular weight polyethylene (UHMWPE) in the 1980s and 1990s [78, 144]. More recently, awareness of the detrimental effects of prolonged shelf-life, problems arising through gamma sterilisation in air and post-sterilisation oxidation and degradation have been recognised and addressed through changes in the sterilisation process [28, 87, 114].

### Patellar fracture

Patella fractures following patellar resurfacing are generally rare, with reported figures ranging from 0.5 to 5.2 % [17, 48, 49, 90, 98, 116]. Although such fractures may result from trauma or from a complication during primary or revision surgery, the majority appear to occur spontaneously [65, 90, 129]. A compromise in patellar vascularity through medial arthrotomy combined with lateral retinacular release is thought to be a major factor in the aetiology of patellar fractures but its clinical significance remains unclear. Some series have demonstrated a relationship between avascularity and fracture [23, 64, 116], whilst others have failed to do so [41, 100, 115]. The literature conveys an array of other potential aetiological factors including technical errors (e.g. patellar mal-tracking secondary to implant mal-alignment, excessive or asymmetric patellar bone resection, thermal necrosis through cement polymerisation), patient demographics (e.g. male gender, obesity with BMI > 30 kg/m^2^, knee flexion beyond 95°, high activity level), and implant design (e.g. large patellar component ≥37 mm in diameter, inlay patellar design, large central fixation peg, posterior stabilising implant) [26, 34, 65, 80, 90, 98, 133, 149].

### Patellar implant loosening

Loosening of the patellar component with or without displacement is reported to occur in 0.6–4.8 % of cases [17, 31, 90]. The frequency of patella component loosening has decreased significantly since the withdrawal of metal-backed patella components in the early 1990s which were notorious for developing wear and loosening [8, 78, 144]. Meding et al. [90] reviewed 8,531 total knee arthroplasties and recorded radiographic evidence of patella component loosening in 409 (4.8 %) cases at a mean of 7 years. In this series, obesity placed the patella at 6.3 times the risk of loosening, followed by lateral release at 3.8 times, elevated joint line at 2.2 times, and flexion beyond 100° at 2.1 times. Other factors identified included poor remaining bone stock, asymmetric patellar resection, small fixation pegs, inadequate implant fixation, patellar mal-tracking secondary to component mal-alignment, osteonecrosis and osteolysis [9, 79].

### Patellar implant wear

**Fig. 5:**
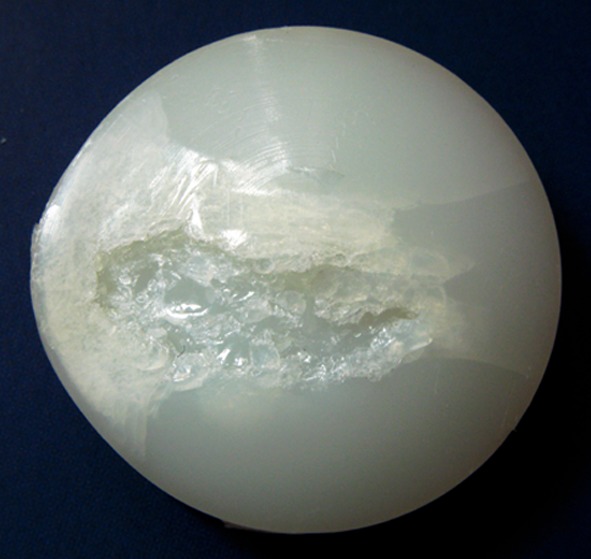
Retrieved patellar component showing signs of catastrophic wear characterised by a variety of wear mechanisms including cold flow, pitting, abrasion, sub-surface fracture, and delmination

Wear is a common feature in patellar implants due to the unfavourable mechanical environment of the patello-femoral articulation [27, 33, 60]. The in vivo wear pattern of patellar implants is highly dependent on the inherent mechanical properties of the materials used (e.g. polyethylene, methylemethacrylate bone cement), the interaction between patella and femoral component, and the external forces acting on them. The mechanical performance of the various designs is best assessed from observations made on retrieval components, which have shown considerable degree of wear and deformation (Fig. 5) [33, 40, 59, 89]. The level of wear damage appears to increase with patient’s weight, the post-operative range of motion, and the length of time the component has been implanted [40]. It is therefore of interest to note that despite patello-femoral compression forces exceeding the yield strength of UHMWPE, catastrophic wear or component fracture are seen infrequently and have not become a significant or endemic problem [146].

### Patellar instability and dislocation

Patellar instability represents a serious problem in TKA and is responsible for a number of associated complications making it the most common reason for secondary surgery including revision [17, 24, 93]. The condition may occur in cases with and without patellar resurfacing, but is more commonly associated with the use of a patellar component. These patients often present with a plethora of symptoms, ranging from mild discomfort to pain, weakness, giving way and locking. Pavlou et al. [104] suggested patellar resurfacing in all cases where satisfactory soft tissue balance cannot be achieved, based on the ill-advised belief that resurfacing as such might overcome minor degrees of mal-tracking. The resurfaced patella, however, carries most probably a higher propensity to emphasise any mal-tracking, whilst the native patella offers at least a limited ability to adapt to adverse conditions over time [69].

The effect of implant design on patello-femoral stability is well recognised [143, 153]. Femoral components featuring a shallow and symmetric trochlea groove with abrupt changes in sagittal radius have been shown to create abnormal patellar kinematics and increase the risk of patellar mal-tracking [24, 106, 148, 158]. Campbell et al. [24] reviewed 289 knee arthroplasties with a shallow and narrow trochlea and found that out of 20 revisions 14 were required for patellar mal-tracking.

Surgical improprieties during patellar resurfacing are common reasons for patellar instability and include residual valgus limb mal-alignment, patella alta, increased internal rotation of femoral or tibial component, medial translation of the femoral component, excessive valgus alignment of the femoral component (even if the overall limb alignment appears neutral), asymmetric patellar resection, lateral placement of the patellar button, excessive patellar composite thickness, improper soft tissue balancing, and failure to perform a lateral release when required [16, 17, 24, 48, 92, 106, 112, 113].

## The unresurfaced patella

**Fig. 6:**
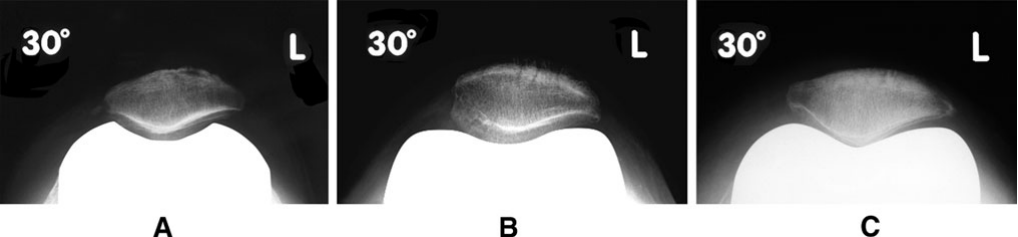
Post-operative skyline radiographs showing the native patella articulating with three different prosthetic femoral TKA components displaying varying degrees of ‘patella-friendly’ design features. A: Optetrak^®^, Exactech, USA; B: AGC^®^ Biomet, USA; C: LCS^®^, DePuy, USA

**Fig. 7:**
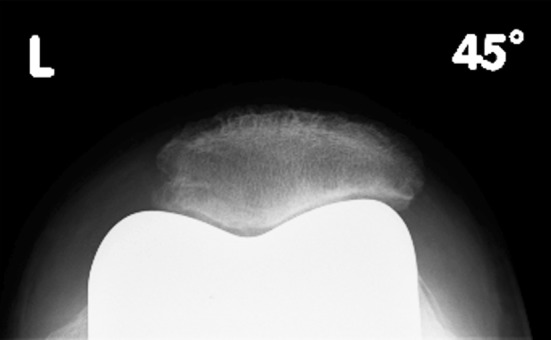
Skyline radiograph obtained 3 years following TKA demonstrating signs of biological remodelling (‘stress contouring’) of the retro-patellar surface

Following bicompartmental knee arthroplasty, the non-resurfaced patella becomes exposed to the metallic surface of the femoral component (Fig. 6). Due to differences in modulus of elasticity, the articular surface of the patella must adapt to the geometry of the opposing surface by bedding in [69]. The process of biological remodelling, also described as ‘stress contouring’, produces a gradual adaptation of the retro-patellar surface and subchondral bone plate to the trochlea shape (Fig. 7) [140]. Keblish and Greenwald noted that minimal remodelling was required if the patella was exposed to an anatomical design with constant radius of curvature and uniform femoral geometry, whilst excessive remodelling was observed in non-anatomical designs [69]. The remodelling process was time dependent and not displayed through axial radiographs much before 2 years after implantation.

Tanzer et al. [147] looked at the effect of femoral component designs on the contact and tracking characteristics of the unresurfaced patella in TKA. The authors noted substantial alterations in patello-femoral contact areas, contact pressures and tracking at higher flexion angles when the native patella was articulating with a prosthetic femoral component. Although the percentage of patello-femoral contact area compared with the native knee reduced markedly with increasing knee flexion, with measured values of 79 % at 60°, 69 % at 90° and 65 % at 105°, it remained well above those measured for the prosthetic patella.

**Fig. 8:**
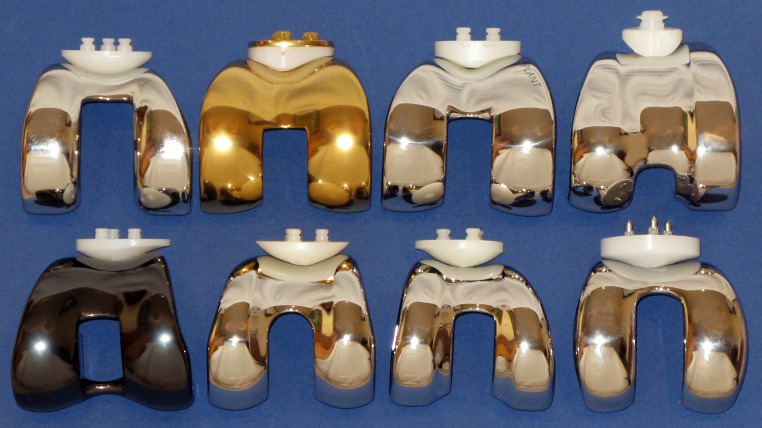
Various femoral arthroplasty components with their respective, designated patellar implant. *Top row*, *left* to *right*: AGC^®^ (dome patella), Biomet, Warsaw, USA; Buechel-Pappas (uncemented anatomic rotating platform patella), Endotec, Orlando, USA; LCS^®^ (anatomical fixed bearing patella), DePuy, Warsaw, USA; Medial rotating knee^®^ (cylindrical patella), Finsbury, England. Bottom row, left to right: Journey^®^ (off-set dome patella), Smith and Nephew, Andover, USA; PFC-Sigma^®^ (modified dome patella), DePuy; Triathlon^®^ (off-set dome patella), Stryker, Kalamazoo, USA; BioPro^®^ Townley Total Knee Original (uncemented metal-backed dome patella), Biopro, Port Huron, USA

The surface geometries of some prosthetic femoral components, particularly those of posterior stabilised design, appear incompatible with the native patella, as the apex of the retro-patellar ridge may impinge on the prosthetic intercondylar notch at knee flexion angles beyond 90° (Fig. 1). Patella deformation and wear are likely consequences, and in the case of significant patellar tilt, displacement of the patella into the notch becomes possible [88]. Distal extension of the trochlea and shortening of the intercondylar notch have been shown to safeguard patellar support beyond 90° of knee flexion [158] (Fig. 3). Such design modifications are hence important if one considers leaving the patella unresurfaced [81]. Most current femoral components, however, present a surface geometry designed to articulate with a designated patella component but are ill equipped to accommodate the native patella [81] (Figs. 6, 8). Specific efforts are required to improve patella kinematics by creating a femoral component which conforms to the normal trochlea and intercondylar notch topography and which takes the geometry of the native patella into account [154]. Only then would we be in a position to offer prostheses dedicated to articulate against the native patella, compared with the mostly inadequate femoral designs available to date.

## Anterior knee pain in TKA

Early arthroplasty designs were particularly prone in causing post-operative AKP as they failed to provide an appropriately shaped articulating surface for the native patella [23, 64, 110, 141]. Despite advances in engineering, modern TKA designs continue to show a wide variations in the incidence of AKP, with reported figures of 0° to 47 % in patients with patellar resurfacing [13, 19, 25, 39, 151], and of 0° to 43 % in those patients where the patella is retained [13, 19, 38, 55, 67, 75, 97, 107, 151, 157]. These variations are likely to be due to differences in pain assessment, patient selection, surgical technique and implant design. Scott and Kim indicated that regardless of the management of the patella, clinicians can expect approximately 10% of patients to be affected by significant AKP after TKA, a finding, which has been confirmed through prospective, observational studies [5, 14, 35, 58, 130].

A significant number of clinical studies have shown that patients undergoing patella resurfacing are less likely to be affected by AKP and overall more satisfied [13, 23, 36, 67, 101, 126, 151]. However, the issue whether patients with non-resurfaced patellae really suffer more pain compared with those who have been resurfaced remains a controversial one. Robertsson et al. [117] reviewed data of 27,372 patients from the Swedish Knee Register and found that 15 % of patients with resurfaced patellae were generally dissatisfied, compared with 19 % where the patella had been retained. However, patients with patellar resurfacing became less satisfied with their knee over time, whilst satisfaction rating in those without resurfacing remained unchanged. The authors concluded that the benefit of the patellar component diminishes with time and that the need for secondary resurfacing may in the longer term be balanced by the need for revision of failed patellar components [117, 118]. A recent meta-analysis of 7,075 cases found no difference regarding the incidence of AKP between resurfacing and non-resurfacing group, which invited the authors to the conclusion that the rate of re-operations in non-resurfaced patients might be artificially increased as secondary resurfacing provides the only viable surgical option for this group of patients [104].

The great debate about the pros and cons of patellar resurfacing revolves around our lack of understanding why, irrespective of pre-operative symptoms and patellar resurfacing, some patients may suffer AKP following TKA and others may not [7, 105]. Even though many clinicians believe that in the presence of pre-operative symptoms resurfacing should be considered, the scientific basis for such action is missing, as no conclusive evidence currently exists. In a randomised controlled trial, Barrack et al. [7] found that 28 % of patients without AKP before resurfacing suffered AKP after surgery. Likewise, 9 % of patients with pre-operative AKP continued having pain post-operatively despite resurfacing. In the group where the patella was retained, 23 % continued suffering pain, whilst new pain developed in 14%. Hasegawa and Ohashi followed 78 unresurfaced TKAs for 12 years. Seventeen (22 %) knees developed patella subluxation and lateral facet erosion, but only four of these (5 %) experienced pain [55].

In many ways, it is erroneous to attribute all AKP to the patella, as a variety of conditions may be responsible for the development of discomfort projected in and around the patello-femoral articulation. Soft tissue afflictions (e.g. peri-patellar tendinopathy, bursitis, impinging synovial folds and scar tissue bands, neuromas, Sudeck dystrophy, complex regional pain syndrome), bony abnormalities (e.g. Sinding-Larson-Johansson syndrome, stress fracture, retained osteophytes, impinging loose bodies), and patellar mal-tracking have all been implicated as potential causes of AKP [18, 19, 124]. Any underlying condition should hence be addressed before treatment is focussed on the patello-femoral articulation.

## Predictors of anterior knee pain

A variety of predictors for post-operative AKP have been suggested but few, like obesity and flexion contracture, have been reliably identified [57, 107, 139, 141]. Most clinical studies have failed to depict differences between knees affected by AKP and those which are not [7, 25, 139, 151]. Insall was unable to define a correlation between the degree of cartilage damage and the level of pain or quality of result in patients who had been left unresurfaced [63, 64, 141]. Elson and Brenkel prospectively assessed 602 primary TKAs and found mild pain in 8 % and moderate to severe pain in 5 % of knees [35]. In their study, age was the only reliable predictor of pain, with patients below the age of 60 being more than twice as likely to be affected. Results from randomised controlled trials have failed to show any association between obesity, pre-operative AKP, degree of chondromalacia or chondrolysis, lateral release and the occurrence of post-operative AKP [7, 25, 139]. Recently, height and weight but not BMI have been delineated as being predictive of anterior pain and of revision in resurfaced patellae, which is thought to be due to increased leaver arms and raised patello-femoral forces displayed in taller and heavier individuals [19, 90, 156]. Rodriguez-Merchán and Gómez-Cardero prospectively reviewed 500 patients without patellar resurfacing whose retro-patellar cartilage had been graded intra-operatively according to Outerbridge’s classification [121]. After a minimum follow-up of 5 years, 11.6 % of patients with grade IV changes required secondary resurfacing compared to 0.6 % of those with grade I–III. The authors concluded that patients with advanced levels of cartilage degradation should be resurfaced at index procedure. In comparison, Barrack et al. [7] found that neither obesity, nor the degree of patellar chondromalacia, or the presence of pre-operative anterior knee pain predicted post-operative clinical scores and the presence of post-operative AKP. Waters and Bentley assessed 514 knees randomised for patellar resurfacing and found no difference between knees with AKP and those without regarding age, weight, gender, lateral release, cruciate retention or sacrifice and whether the knees were affected by osteoarthritis or rheumatoid arthritis [151].

Despite resurfacing or non-resurfacing of the patella, the prevalence of AKP remains high. Combined with the fact that such pain often fails to respond to secondary resurfacing is suggestive that underlying patient, implant or surgical factors, other than patellar resurfacing, may have a significant impact on the presence of AKP following TKA [7, 39, 62]. Figgie et al. [39] were able to show that AKP was present in 23 of 75 TKAs in which the implants were positioned outside the ideal alignment compared with no cases of AKP in the group of 41 knees where components were positioned correctly.

Circumferential thermocoagulation of the patellar rim with electrocautery, which is thought to create a level of sensory deprivation, was first suggested by Keblish in 1991 in an attempt to reduce the likelihood of post-operative AKP when retaining the native patella [68, 69]. Keblish used the procedure in conjunction with debridement and occasionally added transcortical Pridie drilling to areas of cartilage loss. Overall, the scientific literature on the subject is sparse and potential merits of such surgical intervention whether used in conjunction with patellar resurfacing or not remain unclear [52, 77, 105].

Implant design is known to impart a major effect on patella kinematics and it is therefore not inconceivable that such an effect may influence the development of post-operative AKP [55, 106, 147, 158]. The majority of femoral components available today are designed to articulate with their designated patellar prosthesis (Fig. 8). Articulation between native patella and prosthetic femur may induce potential problems in terms of abnormal contact and tracking characteristics [72, 147, 148, 154]. It has hence been speculated that AKP in patients where the patella has been left unresurfaced may be secondary to altered patellar biomechanics and poor femoral component design [13, 84, 85, 140].

How important design issues are has been highlighted by a group of researchers from the University of Western Australia, who conducted two randomised controlled studies with almost identical study design where the only major variable was the type of prosthesis used. In the first study conducted by Wood et al. [156], a relatively unfriendly patellar design, featuring flat-shaped condyles with a shallow and angular trochlea groove was employed. In their second study led by Smith et al. [138], a relatively patellar-friendly design, characterised by a deepened trochlea groove with curved transition toward the femoral condyles was used. Comparing the outcome of non-resurfaced patients between both studies revealed a drop in the rate of post-operative AKP from 31 to 21 %, a reduction in the re-operation rate for patello-femoral complications from 12 to 1.2 %, and an increase in Knee Society Rating Score by 11 points. The group of Beverland examined 10-year data of 600 unresurfaced TKAs utilising an anatomically shaped ‘patella-friendly’ femoral component [97]. The authors found significant AKP leading to secondary resurfacing in only 1.5% of cases and concluded that leaving the patella unresurfaced does not adversely affect the outcome when using a patella-friendly design. Hwang et al. [61] who compared 7-year results of two groups of patients who received a femoral component with patella-friendly design features were unable to detect any significant differences in terms of AKP, or revision rate between resurfaced and unresurfaced knees. A recent review study failed to observe an association between clinical outcome and prosthetic design, but the inclusion criteria used in qualifying ‘patella-friendliness’ were somewhat indiscriminate, resulting in most implants falling into this category [104].

On the basis of our current knowledge, reported results from clinical studies should probably be viewed as being design specific and reliable only for the implant studied. Some older and often retrospective studies have featured implant designs which have either been altered or discontinued, hence substantially impairing their validity. However, despite proper patient and implant selection and good surgical technique, the inability to determine with any degree of certainty, whether a patient may be affected by AKP if the patella is left unresurfaced remains a surgical conundrum and demands further investigations.

## Secondary resurfacing

The number of patella-related revisions is higher if the patella is left unresurfaced and is thought to reflect the higher incidence of AKP in patients with patellar retention. Insertion of a patella component or ‘secondary resurfacing’, considered a remedial procedure to address AKP, is performed in up to 13% of cases [7, 13, 36, 107, 141]. In 1998, Insall conveyed that in his series of several hundred TKAs (IB-II^®^, Zimmer, Warsaw, USA), which was not a particularly patellar-friendly femoral component design, the rate of secondary resurfacing was approximately 8% [66]. In a significant proportion of these patients, however, symptoms are likely to remain unchanged despite secondary resurfacing or revision arthroplasty [94]. Satisfactory outcomes following secondary resurfacing have been reported in 30% to 80% of cases [7, 24, 45, 72, 83, 94, 102, 117, 142]. However, even if the secondary resurfacing procedure appears successful at first, recurrence of symptoms has been reported in up to 55% of patients [7]. In a recent retrospective study, Parvizi et al. [102] reviewed 39 patients at an average of 4.5 years following secondary resurfacing for AKP and encountered 8 patients who expressed their dissatisfaction with the outcome of surgery. However, 14 patients showed no improvement or deterioration in clinical outcome and 7 patients required further revision, with one for mal-tracking of the patella.

Spencer et al. [142] reviewed 28 patients who had undergone secondary patellar resurfacing for persistent AKP. Patient satisfaction was assessed at a mean of 28 months post-operatively, resulting in 59 % feeling improved, 34 % feeling the same and 7 % feeling worse. In a similar study, Garcia, Kraay and Goldberg reviewed 17 cases of isolated patellar resurfacing, of which 53 % were asymptomatic and satisfied, whilst 47 % continued to be affected by AKP and unsatisfied [45]. It would hence appear reasonable to suggest that failure of patients to improve following secondary resurfacing may point to either a multifactorial aetiology or a different cause for pain other than a problem pertaining to the PFJ.

Three-phase bone scintigraphy as an assessment tool to distinguish patients who are likely to benefit from secondary resurfacing has recently been suggested [3]. Increased tracer uptake of the patella in patients with localised AKP appeared predictive of symptomatic pain relief following secondary patellar resurfacing, but overall numbers were small; hence, further research is needed before a principle may be established.

If a patient with a non-resurfaced patella presents with AKP, secondary resurfacing despite its limited success remains an available option and potential remedy. Conversely, there are fewer options available for the treatment of those patients with AKP whose patella has already been resurfaced. Isolated patella component revision for pain is generally not recommended as the clinical outcome is uncertain [10]. Furthermore, patella revision is far from being an innocuous procedure and should be approached with utmost caution as complications are frequent and outcomes poor [10, 74]. It could therefore be argued that if this clinical situation occurs where a patient is affected by AKP following primary patellar resurfacing, the surgeon is less likely to proceed with a revision procedure, which to some extent would explain the higher proportion of revisions in non-resurfaced knee arthroplasties.

Revisions for patello-femoral symptoms are mostly performed relatively soon after the index procedure, whilst revisions for wear or loosening of the patellar implant usually occur much later on. Putting this in perspective with the finding that patients who had their patella resurfaced are at least initially more satisfied with their knee, one might suggest a more liberal use of patellar resurfacing, at least in the elderly population [117, 118, 120].

## National arthroplasty registers

**Fig. 9:**
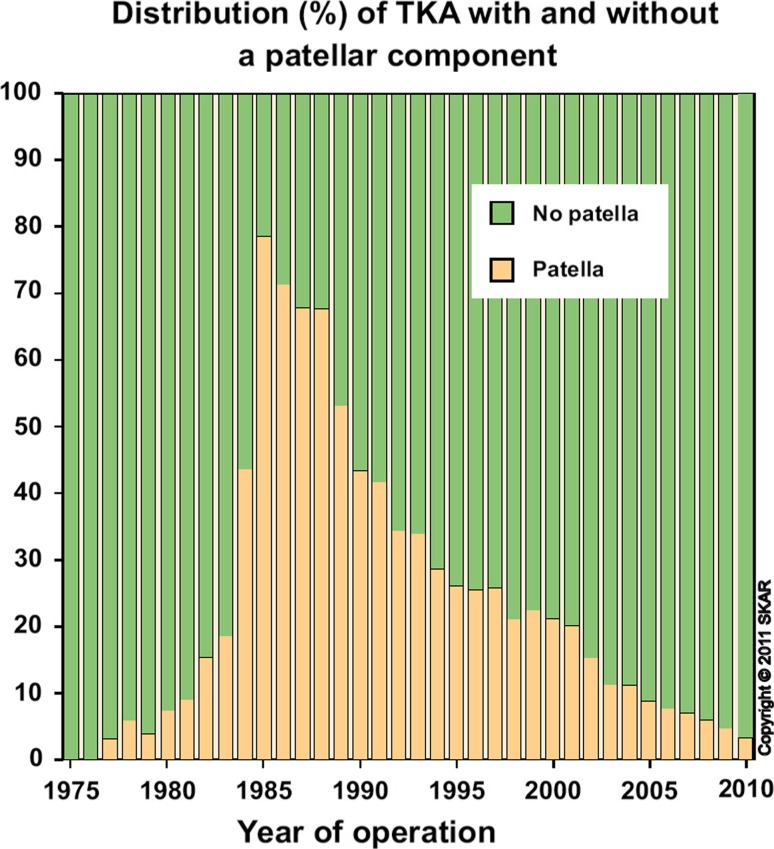
Illustration extracted from the 2010 annual report of the Swedish Knee Arthroplasty Register showing the yearly distribution concerning the use of patellar components in TKA between 1975 and 2010 (Courtesy of Otto Robertsson and with kind permission of the Swedish Arthroplasty Register)

National joint registers are a valuable source of information as it pools data on a large number of patients. Unfortunately, data collection is of variable quality and does not cover all aspects of treatment and complications surrounding the management of the PFJ in TKA [118]. The frequency of implanting a patellar component varies greatly between countries. The Swedish Knee Arthroplasty Register has provided long-term data on the use of patellar components in TKA since 1975 [145]. Following a peak in patellar resurfacing during the 1980s, with rates of over 70 %, there has been a steady decline in the number of TKA receiving a patellar component (Fig. 9). In the most recent report published in 2010, patella resurfacing as part of a TKA was performed in just over 3% of cases [145]. Although the register revealed a higher rate of revision in unresurfaced TKAs, the difference was not statistically significant.

**Fig. 10:**
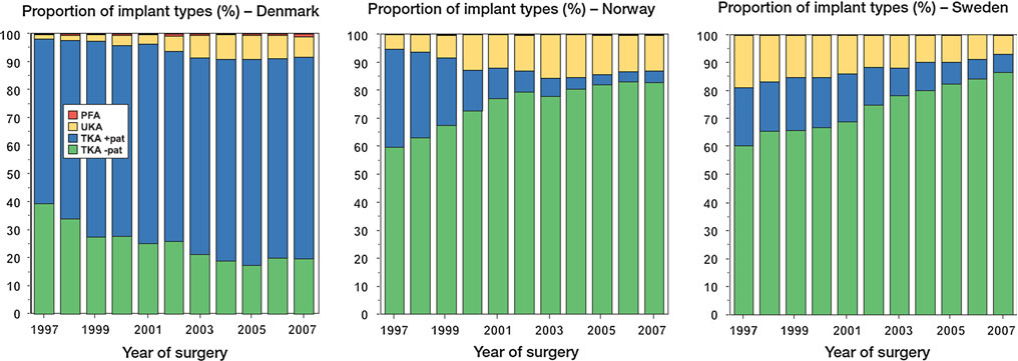
Proportion of implants types used for primary knee arthroplasty in Denmark, Norway and Sweden. *Blue column* demonstrates the proportion of resurfaced patellae, and *green column* demonstrates the proportion of patellae which have been left unresurfaced. Please note the significant differences and trends regarding patella resurfacing between the three countries [119]. Courtesy of Otto Robertsson and with kind permission of Acta Orthopaedica)

In comparison, data from the 2009 arthroplasty register report in Norway indicated that out of a total of 3965 TKAs, only 96 (2.4 %) received a patellar component, whilst secondary resurfacing for AKP was performed in 1.8% of all arthroplasty cases [44, 96] (Fig. 10). According to the 2010 annual report of the Danish Knee Arthroplasty Register, it was estimated that the use of patellar resurfacing in TKA had increased from 68 % in 1997–2000 to 80 % in 2009 [29] (Fig. 10). The report further revealed that of all revision procedures performed in Denmark, 9.1 % are performed for secondary patellar resurfacing and 5.1 % for polyethylene wear of patellar components. Reported figures from the 2011 Annual Australian National Joint Replacement Registry Report confirmed an increase in the rate of resurfacing from 41.5 % in 2005 to 49.5 % in 2010 [6]. If the patella was left unresurfaced, the cumulative revision rate for posterior stabilised implants at 10 years was calculated at 8.1 %, compared with 5.8 % for all others. Patello-femoral pain was listed as the reason for revision in about 13.5 % of all primary TKAs. Interestingly, the Australian figures show significant variations in the usage of patella components between States and Territories.

Robertsson et al. [119] recently analysed 10-year data from the Nordic Arthroplasty Association obtained between 1997 and 2007. To the authors it remained unclear why the use of patellar components increased in Denmark but decreased in Norway and Sweden in the given time frame and why surgical practice in these counties differs so significantly (Fig. 10). It is unlikely that the variations in the proportion of resurfaced primary patellae between National joint registers can be attributable to cultural differences alone. It may hence be assumed that surgeon’s choices must have been affected by clinical evidence, experience, education, tradition and manufacturers marketing politics or a combination thereof [120].

## Prospective and randomised controlled trials

### Unilateral trials

The controversy surrounding the need for patellar resurfacing at the time of TKA has been fuelled by differing results derived from clinical studies and historic data. Unfortunately, most studies are retrospective and utilising redundant implant designs. They are often inadvertently affected by observer bias and their methodological limitations prevent a direct comparison of like-for-like. These studies have henceforth done little to reduce the insurmountable divide between clinicians who promote resurfacing and those who do not. Randomised, controlled, prospective trials have tried to address these shortcomings, but variations in patient assessment and study design remain and continue to impair their comparability.

A meta-analysis of 16 randomised controlled trials (RCT) revealed a total of 1,587 knees which were treated with patellar resurfacing at the time of TKA, compared with 1,620 knees where the patella was left unresurfaced [7, 15, 19, 21, 25, 37, 38, 46, 77, 95, 101, 126, 139, 151, 156] (Table 1). The average follow-up period was 5.4 years (range 1–10.8 years). Post-operative AKP was present in 20.8 % of unresurfaced and 16.8% of resurfaced patellae. Knee Society scores of 155 in unresurfaced and 153 in resurfaced patellae were recorded. Patellar complications lead to a reoperation rate of 4.4 % in all unresurfaced and of 2.1 % in all resurfaced patellae. Overall, 9 studies were unable to define a clinically significant difference between resurfacing and non-resurfacing in patients’ function and their perception of pain, two studies showed slight preference towards non-resurfacing, whilst in five studies, resurfacing appeared superior over non-resurfacing.

**Table 1:** Randomised controlled trials published between 1995 and 2011 comparing the outcome of total knee arthroplasty with and without patellar resurfacing

	TKA implant type	Patellar implant type	Number of cases NR/RS	Mean follow-up (years)	NR AKP (%)	RS AKP (%)	NR ROP (%)	RS ROP (%)	NR KSS	RS KSS	Comments
Partio and Wirz [101]	PFC CR	Modified dome	50/50	2.5	22	2	0	0	169	170	RS better
Feller et al. [37]	PCA	Off-set dome	20/20	3	n.s.	n.s.	0	5	(89)*	(86)*	NR better
Schroeder-Boersch et al. [126]	Duracon	Onlay	20/20	4.8	20	10	10	5	150	163	RS better
Barrack et al. [7]	MG-II CR	Modified dome	60/58	5	17	19	12	0	169	162	No difference
Fengler [38]	PFC	Dome (inlay)	68/68	1	0	0	0	0	147	138	NR better
Wood et al. [156]	MG-II CR	Not specified	128/92	4	31	16	12	10	152	157	RS better
Waters and Bentlely [151]	PFC CR/PS	Dome	231/243	5.3	25.1	5.3	4.8	1.2	162	167	RS better
Burnett et al. [19]	AMK CR	Dome	48/42	10.8	25	37	6	2	146	145	No difference
Gildone et al. [46]	NexGen PS	Dome	28/28	2	21	0	0	0	178	178	RS better
Myles et al. [95]	LCS RP	Anatomic	25/25	1.75	n.s.	n.s.	0	0	162	147	No difference
Campbell et al. [25]	MG-II CR	Modified dome	54/46	10	43	47	3.7	2.2	136**	138**	No difference
Burnett et al. [20]	MG-II CR	Modified dome	32/32	10	17.3	16.5	6.2	3.1	148	146	No difference
Smith et al. [139]	Profix	Dome (inlay)	86/73	4.4	21	30	1.2	1.4	163	152	No difference
Burnett et al. [21]	MG-II CR	Modified dome	60/58	10	16	21	12	3	155	146	No difference
Liu et al. [77]	PFC—PS	Modified dome	64/68	7	12.5	14.7	0	0	125	121	No difference
Breeman et al. [15]^‡^	Multiple	Multiple	646/664	5	n.s.	n.s.	2.4	1.3	(34.0)^†^	(35.1)^†^	No difference
Total			1,620/1,587	5.4	20.8	16.8	4.4	2.1	155	153	

*NR* not resurfaced, *RS* resurfaced, *n.s.* not specified, *AKP* anterior nee pain, *ROP* reoperation rate, *KSS* knee society rating score

* HSS rating score used, ** 4 year follow-up data only, ^†^ Oxford knee score, ^‡^ multi-centre trial

Some of these studies have examined knee function in more detail by assessing the patient’s ability to climb stairs [19, 25, 37, 46, 139, 156]. Bourne et al. [11] who devised a 30s stair climbing test found no statistically significant difference at 2-year follow-up between patients with and without patellar resurfacing. The same group of patients was again reviewed at 10 years, by which time those with patella resurfacing climbed on average 20 stairs compared with 31 stairs in the non-resurfaced group, a difference which reached statistical significance [19]. Similar findings were reported by Feller et al. [37] who found that the stair climbing ability in the non-resurfaced patient group was significantly better compared with those with patella resurfacing. Two RCTs found no significant difference regarding the performance of functional tasks between resurfaced and non-resurfaced patients [46, 139], whilst two other RCTs showed a trend toward increased pain with stair ascend and descend, although values did not reach statistical significance [25, 156].

Two randomised controlled biomechanical studies looked at functional range of movement and walking gait pattern [95, 138]. Both studies were unable to delineate any clinically relevant differences between resurfaced and non-resurfaced knees, but highlighted discrepancies in kinematics compared with normal individuals.

### Bilateral comparative trials

A total of 10 studies (prospective or randomised controlled) incorporating a comparative assessment of patients who received bilateral total knee arthroplasties, with patellar resurfacing performed on one side only, were identified [7, 20, 36, 69, 76, 103, 105, 135, 139, 151] (Table 2). A meta-analysis of these studies revealed a total of 299 patients, who had been followed-up between 2 and 10 years (average 5 years). Satisfaction was assessed by asking patients which knee they prefer. The resurfaced side was favoured by 35 % of all patients, the non-resurfaced side by 18 %, and 47 % expressed no preference for either knee.

**Table 2:** Randomised and prospective trials published between 1989 and 2011 where patients received bilateral total knee arthroplasties with the patella being resurfaced on one side only

	TKA type	Patellar implant type	Type of trial	Number of cases	Mean follow-up (years)	RS preferred (%)	NR preferred (%)	No preference (%)	Author’s comments
Shoji et al. [135]	Yoshino-Shoji total condylar CS	Not specified	Prospective	35	2	23	29	48	Routine resurfacing not advisable
Enis et al. [36]	Townley	Dome metal backed	Prospective	20	3.3	45	15	40	Better pain relief with resurfacing
Levitsky et al. [76]	Not specified	Not specified	Retrospective	13	7.5	46	8	46	Patellar retention acceptable if selection criteria applied
Keblish et al. [69]	LCS RP	Anatomic RP	Prospective	30	5.2	30	23	47	Patellar retention acceptable with patella-friendly implant
Barrack et al. [7]	MG-II CR	Modified dome	Randomised	23	5	21	29	50	Anterior knee pain unrelated to patellar resurfacing
Waters and Bentley [151]	PFC CR/CS	Dome	Randomised	35	5.3	51	11	37	Patellar resurfacing preferred
Peng et al. [105]	NexGen/MG-II	Dome	Prospective	35	3.2	28	26	46	No difference
Burnett et al. [20]	MG-II CR	Modified dome	Randomised	32	10	37	22	41	Equivalent clinical results
Smith et al. [139]	Profix	Dome (Inlay)	Randomised	16	4.4	–	–	100	No benefit of patellar resurfacing over non-resurfacing
Patel and Raut [103]	PFC	Modified dome	Prospective (staged)	60	4.5	68	15	17	Resurfacing recommended. Secondary resurfacing in 4 patients
Total				299	5	35	18	47	

*NR* not resurfaced, *RS* resurfaced

## Conclusion

The patella represents an integral part of any TKA and clinicians must be aware that the surgical management of the patella will not only affect patient satisfaction but occupies a pivotal role in success or failure of TKA. The appreciation of the consequences of the mechanical environment on the behaviour of the PFJ is of particular importance when contemplating patellar resurfacing. Clinicians should hence possess principle knowledge of anatomy, biomechanics and kinematics of the knee and the locomotor system, as surgically imposed changes may impart significant effects on performance and behaviour of the PFJ [123, 124]. In addition, awareness of the importance of proper component alignment and the effects of mal-positioning on the PFJ are paramount in achieving long-term success, regardless as to whether the patella is resurfaced or not. Surgical technique and implant design have been unequivocally identified as major factors in influencing clinical outcome, and their improvements have helped to reduce the incidence of AKP and patella-related complications.

The orthopaedic community, however, remains deeply divided regarding the issue of patellar resurfacing and the argument for or against continues to be unresolved. Opponents of resurfacing contend that the native patella provides better patellar tracking, improved clinical function, and avoids implant-related complications, whilst proponents of resurfacing argue that patients have less pain, are overall more satisfied, and avert the need for secondary resurfacing. Clinicians have to weigh the possible risk of secondary patella resurfacing for anterior pain against an increased probability of complications arising from patellar resurfacing and future component revision.

The scientific literature can be confusing as it offers as much evidence in support of routine resurfacing as in non-resurfacing. Recent evidence-based research and meta-analysis have failed to draw clear conclusions and therefore have been unable to provide clinicians with specific guidance [12, 22, 43, 56, 90, 104]. It is therefore not surprising that national arthroplasty register data show wide variations in the proportion of patellar resurfacing between countries, reasons for which cannot be accounted for by cultural differences alone and are likely to be multifactorial.

Available randomised controlled trials have so far only considered the ‘all-or-nothing’ approach of always or never to resurface, whilst ignoring ‘selective resurfacing’ as a possible treatment arm. The two standpoints of always to resurface or never to resurface, however, treat the patella indiscriminately based on a random choice. The paradigm of selective patellar resurfacing is attempting to identify those individuals who are thought to have an improved clinical outcome with patellar resurfacing whilst avoiding potential complications associated with unnecessary resurfacing. Selective resurfacing appears as a tempting proposition but evidence regarding the validity of selection criteria remains elusive and the decision when to resurface is often based on intuitive reasoning alone. It is therefore necessary that we define suitable indicators that will tell us who might benefit from a resurfacing procedure, in order to improve the reliability of the selection process. Our endeavours, however, remain hampered by a paucity of validated outcome measures as currently available assessment tools and scoring systems lack sensitivity to detect subtle differences in patello-femoral pain and function. Until we are able to obtain an unambiguous agreement on best practice on patella resurfacing, it may not be unreasonable to consider the compromise of selective resurfacing as middle ground between the two extreme views of always or never to resurface, or in the words of the Roman poet Ovid (43BC-18AD) “*In medio tutissimus ibis*”*.*
